# A sex-adjusted 7-biomarker clinical aging clock for translational preventative medicine

**DOI:** 10.1038/s41598-025-27478-9

**Published:** 2025-12-10

**Authors:** David H. Meyer, Gabriel Mejia, Adrian Molière, Panupong Wangprapa, Tanawat Khunlertkit, Polakit Teekakirikul, Collin Y. Ewald, Claudio Viecelli

**Affiliations:** 1 Keyoniq Technologies AG, Baar, CH-6340 Switzerland; 2 Laboratory of Extracellular Matrix Regeneration, Institute of Translational Medicine, Department of Health Sciences and Technology, ETH Zürich, Schwerzenbach, CH-8603 Switzerland; 3 Bumrungrad International Hospital, Bangkok, Thailand; 4 VitalLife Scientific Wellness Center, Bangkok, Thailand; 5 Heart Institute and Genomic Medicine Institute, Bumrungrad International Hospital, Bangkok, Thailand; 6 University Department of Geriatric Medicine Felix Platter, Basel, Switzerland

**Keywords:** Biological age clock, Preventive medicine, Blood test, Longevity, Healthy aging, Age gap, Ageing, Predictive markers

## Abstract

Biological aging clocks capture heterogeneous rates of aging in individuals and transform current medical practice toward translational preventive medicine. Here, we developed a clinical aging clock based on routine blood biochemistry markers from 59,741 healthy samples in a Southeast Asian cohort. We established a novel correction method to address the systematic skew in predictions from first-generation clocks. This correction improved the accuracy of age-acceleration predictions for disease risks and enhanced interpretability for disease-driven and organ-specific aging processes without relying on mortality data. Based on only seven biomarkers, our clock accurately predicts both self-reported and physician-annotated ICD health data, indicating an increased hazard ratio. Importantly, the clock is robust even in the presence of acute infections or transient immune activation. To demonstrate the multi-ethnic generalizability of our biological age clock, we validated our approach using data from both the NHANES and UK Biobank cohorts. Our approach demonstrates the feasibility of a simple, robust, and interpretable clinical aging clock with potential for real-world implementation in personalized health monitoring and preventive care.

## Introduction

In the developed world, life expectancy has increased significantly in the last hundred years^1,2^. Aging is a major risk factor for developing chronic diseases^3–5^ and a decline in physiological function^6^. This challenges global healthcare systems tremendously, as the costs of chronic diseases are projected to amount to USD 47 trillion by the year 2030^7^. Furthermore, increasing healthcare costs have been shown to be negatively correlated with economic growth, as every 1% increase in the healthcare burden leads to a 0.083% decrease in the GDP growth rate, using cross-country panel data^8^. The slowdown in aging that increases life expectancy by 1 year is estimated to be worth USD 38 trillion^9,10^, highlighting the importance of disease prevention.

Biological age clocks, describing the degree of aging of individuals^11,12^, in contrast to chronological age simply being a function of time passed since birth, hold considerable potential to become key measures for personalized disease prevention^13–15^.

Currently, biological aging clocks for many types of OMICs data are in use, including DNA methylation^16–19^, transcriptomics^20^, lipidomics^21^, and stochastic data^22,23^. While these OMICs data clocks enable highly accurate age predictions, clinical laboratory biomarkers are easier to measure, cost-effective, and often routinely assessed during medical check-ups, making them practical and widely accessible for aging clocks. This simplicity and longitudinal monitoring of biochemical markers in clinical settings make these clocks appealing for a broader application. Early studies attempted to compute the biological age using various clinical and physiological parameters with a multiple regression model^24–26^, showing that worse subjective health is associated with an accelerated predicted age^27,28^. However, these early studies were met with controversy and skepticism, particularly regarding the accuracy of the age predictions^29–31^. In response, more sophisticated methods were developed, such as the Hochschild method^32^ and the Klemera and Doubal method (KDM), which proposes a mathematically optimal way to improve the precision of biological age predictions given several assumptions^33^. Conversely, it was later shown that chronological age predicts mortality to a higher degree than KDM alone^34^. With the advent of deep neural networks, accurate nonlinear blood biochemistry aging clocks have been developed^35,36^. However, despite their reported accuracy, these models often fail to generalize well to other populations^37,38^. They also exhibit a prediction skew, i.e., that younger individuals are systematically predicted to be older, while older individuals are predicted to be younger. This limits their application across diverse groups and age ranges and complicates the interpretation of deviations between the predicted and chronological age.

Second-generation aging clocks aim to improve the accuracy of biological age predictions by training on mortality data rather than chronological age. For example, PhenoAge uses a Cox penalized regression model directly based on mortality data^17^, and more recently, a principal component-based clinical aging clock improved the prediction further^39,40^ by leveraging Nakamura and colleagues’ insights to use principal components to generate uncorrelated variables that reduce systematic biases in the prediction^41^.

Although these mortality-based models outperform those based on chronological age, they are constrained by the limited availability of mortality data, which is often sparse and predominantly derived from Caucasian populations^42,43^. This limitation hampers the development of second-generation aging clocks and their broad applicability, especially in diverse populations.

To address this, we developed a novel correction method that eliminates the systematic prediction skew inherent in previous biological age models, enabling accurate biological age predictions without the need for mortality data. We demonstrate that several diseases show significant biological age acceleration, and importantly, this acceleration is already detectable several years before clinical diagnosis, demonstrating the potential of our clock to capture disease-related aging dynamics. For the first time, a biological age clock has been built using a Southeast Asian dataset, and we show that our clock generalizes well to the ethnically diverse NHANES and UK Biobank cohorts, an important validation as aging clocks have been shown to exhibit systematic ethnicity biases^37,38^. Additionally, we developed a model based on just 7 biomarkers, all routinely obtained at regular check-up intervals, making it practical, inexpensive, and highly predictive across diverse populations.

## Results

**Fig. 1:**
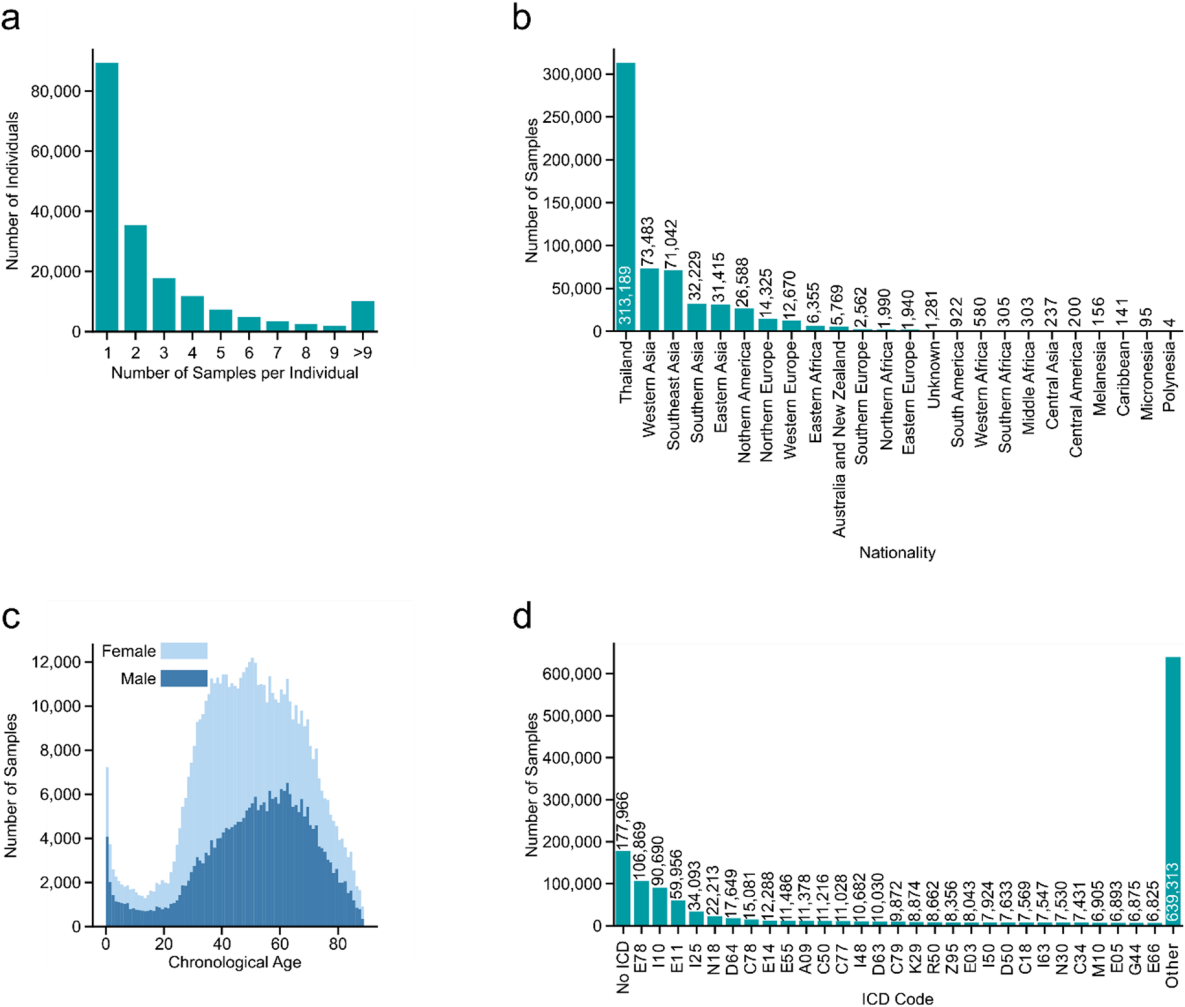
Overview of dataset demographics and clinical characteristics. **(a)** Overview of the number of samples taken per individual, with the majority of individuals being sampled once. **(b)** Number of samples present in the dataset for different ethnicities. The majority of samples come from Thai individuals, with other Asian regions making up the majority of the rest of the samples. **(c)** Age distribution of the samples for female (*light blue color*) and male (*dark blue color*) individuals. **(d)** Frequencies of the most common diseases categorized by ICD codes. The most common recorded diseases are E78 (lipidemias), I10 (essential hypertension), E11 (type 2 diabetes mellitus), I25 (chronic ischemic heart disease), and N18 (chronic kidney disease). Note that individual samples may be annotated with multiple ICD codes.

**Fig. 2:**
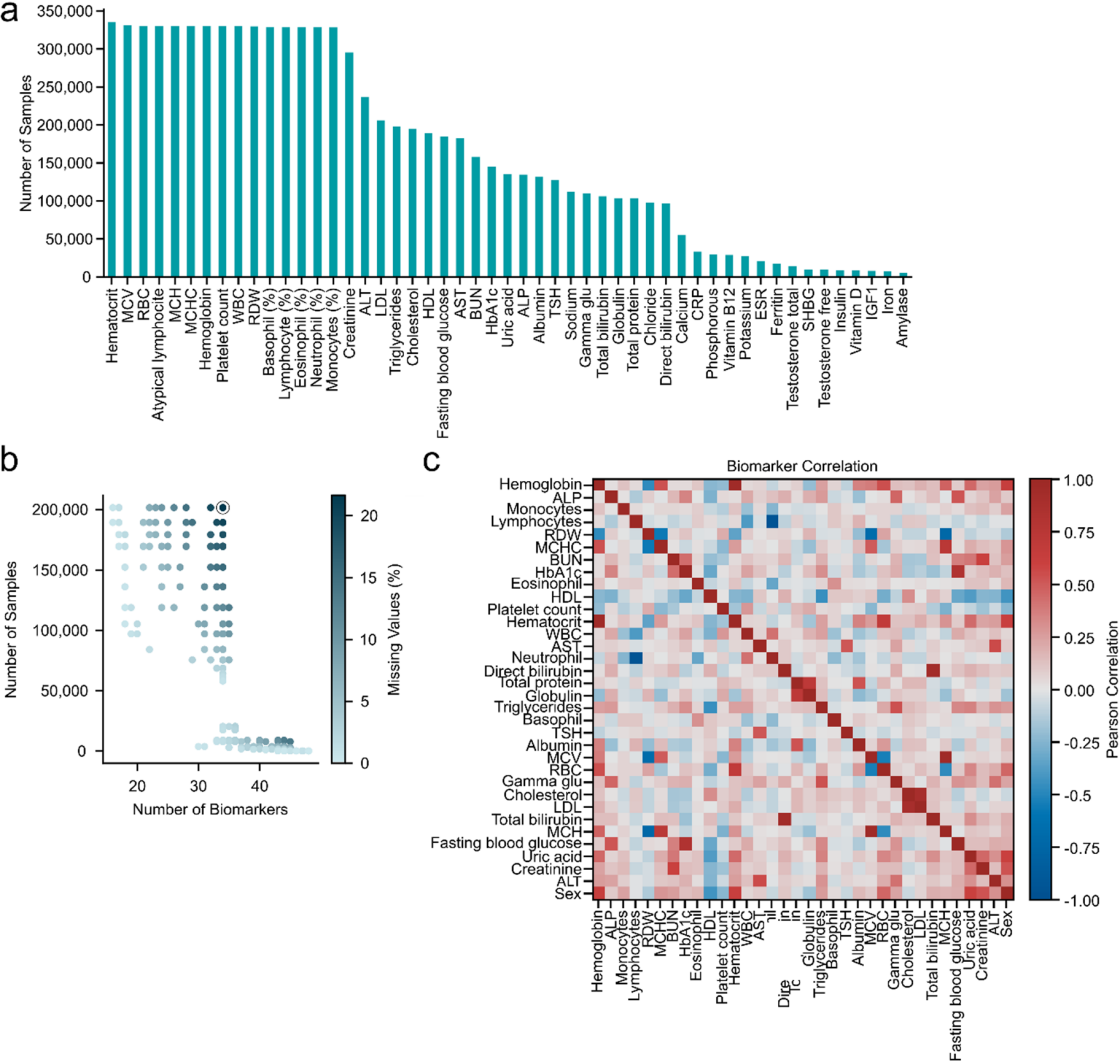
Overview of blood biomarkers. **A)** Overview of measurement frequencies of the 51 blood biomarkers. **b)** Trade-off between biomarker count and sample size. Each dot represents a combination of sample count and number of biomarkers retained, with the color scale indicating the percentage of missing values requiring imputation. The final selection (highlighted by a black circle) includes 34 biomarkers across ~ 200 k samples, with 22% of missing data. **c)** Pearson correlation heatmap of the 34 selected biomarkers.

We used a clinical cohort dataset (see Methods for details) from the Bumrungrad International Hospital (Bangkok, Thailand)^44^, encompassing 184,833 individuals and comprising 597,781 samples from 2000 to 2022 (Supplementary Table 1). Of these, 89,368 individuals (48.3%) had only one blood sample, while 95,465 individuals (51.7%) had at least two blood samples taken (Fig. 1a). A total of 52.4% of the participants were from Thailand, followed by another third from other Asian countries, with a balanced sex distribution and a median age of 49 years (Fig. 1b and c). In this cohort, 70.2% of all samples were associated with one or multiple International Classification of Diseases (ICD) codes, including diagnoses of various conditions, such as disorders of lipoprotein metabolism (E78, 17.9%), hypertension (I10, 15.2%), and type 2 diabetes (E11, 10%), which were the top three diseases (Fig. 1d). A total of 597,781 samples contained data on 174 clinical biochemistry biomarkers. Through expert consensus and iterative refinement, the biomarker set was refined to 51. These 51 biomarkers included blood cell composition and size, as well as creatinine, alanine transaminase (ALT), low-density lipoprotein (LDL), triglycerides, cholesterol, high-density lipoprotein (HDL), and blood glucose (Fig. 2a). The varying frequencies of the biomarkers posed a limitation on the number of representative biomarkers that could be used to build an aging clock (Fig. 2b). To ensure robust data quality, we required each sample to include at least 20 biomarkers and each biomarker to be present in at least 30% of the samples (Fig. 2b). Limiting the imputed data to approximately 22% (see Methods for details) resulted in a significant decrease in the number of usable samples with more than 34 biomarkers measured (Fig. 2b). In other words, there was a trade-off between the number of biomarkers selected and the tolerance for missing values, with the number of usable samples decreasing as more biomarkers were included (Fig. 2b). After applying the filtering steps, 171,812 samples remained. Of these, 59,741 samples from 41,101 individuals were classified as healthy based on the absence of ICD codes and chronic medication usage. The distributions of the 171,812 samples with regard to nationality, sex, age, and ICD codes were similar to those observed in the total dataset (Supplementary Fig. 1a-c). We performed a correlation analysis of these 34 biomarkers across all samples to validate the dataset by verifying known relationships among biomarkers (Fig. 2c). Moreover, identifying strong correlations among biomarkers could indicate redundancy, potentially limiting the aging clock’s ability to capture diverse aging patterns and reducing the interpretability of the resulting biomarker weights (Fig. 2c). Taken together, we selected 34 clinical blood biomarkers to build the first Southeast Asian biological aging clock based on 59,741 healthy samples.

### A skewness correction to improve the prediction of disease age gaps

**Fig. 3:**
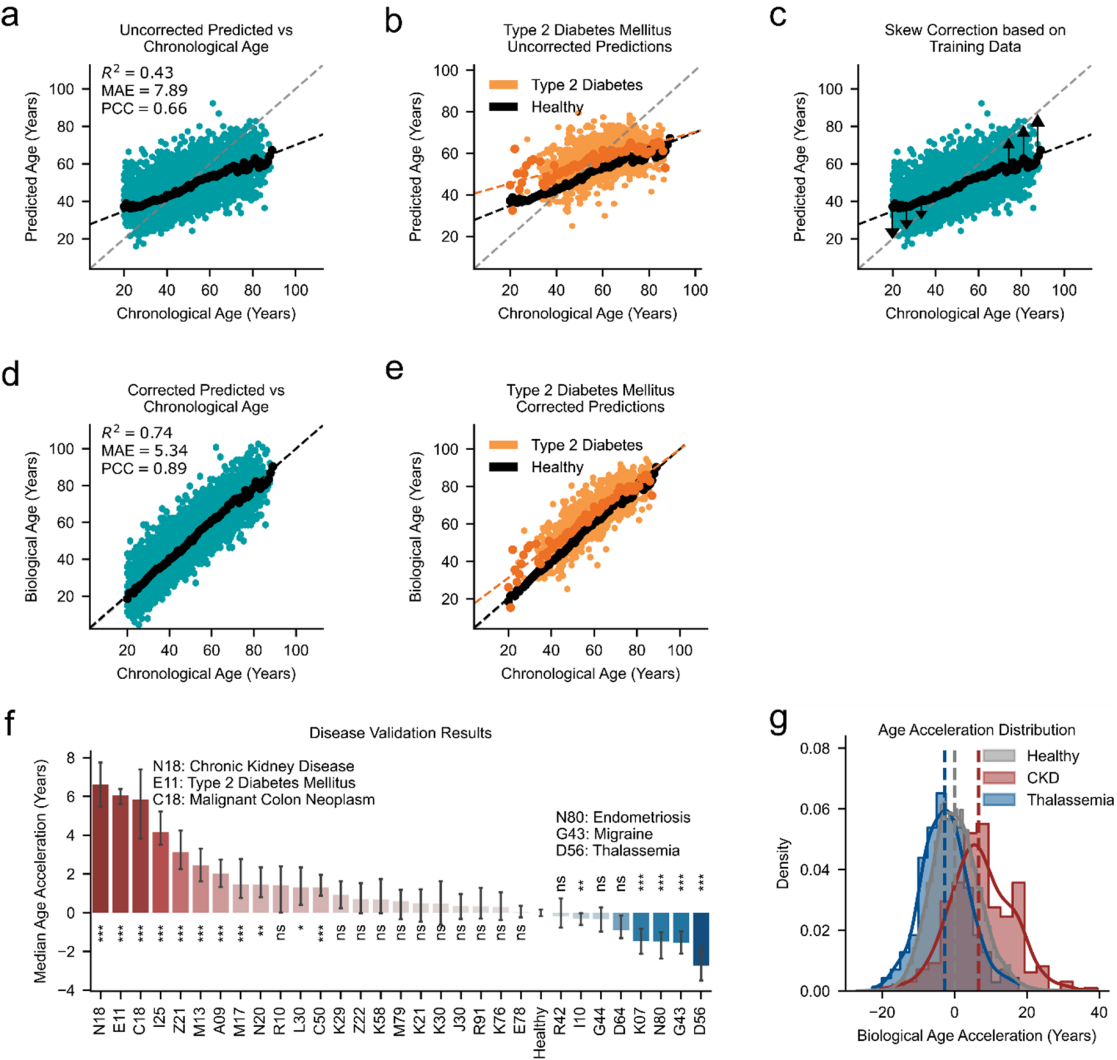
Performance of the 34-biomarker clock, skew correction, and age acceleration in chronic diseases. **(a)** Chronological age vs. predicted age using the 34-biomarker clock with an MLP regressor on the held-out test set. R^2^: 0.43, MAE: 7.89 years, PCC: 0.66. (*black dots*) indicate the median predicted age per chronological age bin among healthy individuals. **(b)** Type 2 diabetes samples (*orange color*) were consistently predicted to be older than healthy samples (*black dots*) with the 34-biomarker clock, independent of their chronological age. The median predicted age per chronological age bin is shown as darker dots for each group. **(c)** The effect of skew correction through the interpolated median predicted age for each chronological age category within the training data is shown, which was used to adjust the predictions in the test and validation datasets, removing the systematic bias. **(d)** Biological age prediction after skew correction: R^2^: 0.74, MAE: 5.3 years, PCC: 0.89. (*black dots*) indicate the median biological age per chronological age bin among healthy individuals. **(e)** Type 2 diabetes samples (*orange color*) are consistently predicted to be older than healthy samples (*black* *dots*), independent of their chronological age, following skew correction. Darker dots show the median biological age for each group at each chronological age bin. **(f)** Median age acceleration for chronic diseases. Most chronic diseases result in an increased predicted biological age, with N18 (chronic kidney disease) resulting in the largest increase. D56 (thalassemia) resulted in the strongest reduction in predicted age. Bars represent medians ± 95% CI. Statistical comparisons were performed using the Mann-Whitney U test with Bonferroni correction for multiple comparisons. All comparisons were conducted against the healthy test group. Adjusted P values are annotated: ns (not significant), *: *P* < 0.05, **: *P* < 0.01, ***: *P* < 0.001 **(g)** Age acceleration distribution for healthy controls (*grey color*), thalassemia (*blue color*), and chronic kidney disease (CKD, *red color*).

Building on this dataset of 59,741 healthy samples, we developed an aging clock using a multilayer perceptron (MLP) regressor^45,46^, trained with a 60/20/20 train/validation/test split of the healthy cohort, while samples classified as unhealthy were reserved for independent validation purposes. The model demonstrated moderate predictive performance, achieving a coefficient of determination (R^2^) of 0.43, a mean absolute error (MAE) of 7.89 years, and a Pearson correlation coefficient (PCC) of 0.66 on the hold-out test data (Fig. 3a). We observed a systematic skew in the model, with younger individuals consistently predicted to be older than their chronological age, while older individuals were predicted to be younger (Fig. 3a). This bias in first-generation aging clocks has been recognized since the inception of the aging clock field^32,47^ and continues in recent publications^36,37^. This generally observed systematic skew reduces the clock’s applicability, limiting the interpretability of the predicted age. Nevertheless, our model successfully captured a meaningful biological age gap, as samples from individuals with type 2 diabetes were consistently predicted to be older than their healthy counterparts, regardless of their chronological age (Fig. 3b). However, the systematic skew hinders the interpretation, as both diabetic and healthy samples at a young age, for example, would be predicted to be older than their chronological age.

To address this skew, we explored several classical strategies, including subsampling to achieve balanced training and applying various output transformations (Supplementary Fig. 2a-e). However, none of these approaches effectively eliminated the systematic skew, and, in some cases, they introduced a more pronounced bias (Supplementary Fig. 2).

To resolve this, we developed a skew correction method (Fig. 3c) to reduce the systematic skew in the predicted age across the age spectrum. Specifically, for each chronological age in the training set, we computed the median predicted age, capturing how much the model tends to over- or under-predict at different ages. We then fit a smooth correction curve to these medians. This curve was used to adjust predictions in the test and validation sets by subtracting the age-specific median offset. This approach, while conceptually similar to age-bias corrections applied in other aging clock domains^48–50^, differs in that it uses a non-parametric smoothing of median prediction errors and strictly applies the correction based on training data only. This ensures robust adjustment without introducing data leakage and effectively centers the predictions along the identity line (see Methods for details). Our correction aligns the predicted ages with the line of identity (Fig. 3d), allowing for a more meaningful comparison of the corrected predicted (biological) and chronological ages. Although this adjustment artificially improves the chronological age predictions, the primary objective of the correction is not to optimize chronological age estimates but to ensure that deviations from the chronological age are biologically meaningful and interpretable. By removing the systematic skew, the correction facilitates the investigation of age-related deviations and their implications for health and disease.

After applying the skew correction, samples from individuals with diabetes (i.e., those already diagnosed with diabetes at the time of blood sampling) continue to be predicted as older than their healthy counterparts of the same chronological age (Fig. 3e), consistent with the uncorrected model (Fig. 3b). However, the correction eliminated the prediction skew, with healthy samples subsequently aligning with the line of identity. By contrast, diabetic samples consistently exhibited a biological age acceleration, defined as predicted ages exceeding the chronological age, as evidenced by their positioning above the line of identity (Fig. 3e). Therefore, the skew correction preserves relative age differences between groups and enables more accurate interpretation by eliminating the systematic skew that previously obscured individual-level comparisons. Similarly, all major chronic diseases (evaluated in individuals with a confirmed and isolated diagnosis at the time of sampling), such as chronic kidney disease (CKD, N18) or chronic ischemic heart disease (I25), demonstrated a significant age acceleration compared to healthy individuals (Fig. 3f). Interestingly, thalassemia (D56), endometriosis (N80), migraine (G43), and dentofacial anomalies (K07) consistently showed a younger predicted age (Fig. 3f, g).

Taken together, by correcting for the systematic skew in the predicted age, our clock achieves greater precision in detecting age acceleration associated with diseases, thereby paving the way for more accurate and interpretable assessments of biological aging.

### A 7-biomarker clock accurately predicts disease age gaps and underlying drivers for diseases

Biological aging clocks offer valuable insights into age acceleration and disease development, making them a very powerful clinical tool for preventive medicine^51^. However, the applicability of biological aging clocks in preventive clinical settings is closely linked to the effectiveness and efficiency of biomarker screening protocols, as well as their economic feasibility. To address this, we investigated the relationship between the performance of the regression model and the number of biomarkers utilized. Our aim was to unlock the full preventive potential of our biological aging clock.

Using an iterative approach, we systematically removed biomarkers with the least impact on predictive performance (see Methods for details), ultimately narrowing the model down to six clinical biochemistry markers plus sex. These six biomarkers were creatinine, hemoglobin A1c (HbA1c), alanine aminotransferase (ALT), high-density lipoprotein (HDL), triglycerides, and albumin.

Creatinine is a byproduct of muscle energy metabolism that is filtered by the kidneys, indicating how efficiently the kidneys work to eliminate waste products from the blood^52,53^. Higher creatinine levels are considered a warning sign of premature aging due to their close association with cardiovascular disease and systemic functional decline^54^.

Hemoglobin A1c is a clinical biomarker that reflects the average blood glucose concentration over the previous 2-3 months^55^. Glycation is a non-enzymatic process in which glucose binds to proteins, forming advanced glycation end products, which accumulate with age and contribute to cellular and tissue dysfunction^56^. Elevated HbA1c is causally linked to accelerated neurodegeneration, cognitive decline, and increased mortality, acting as a surrogate marker for the cumulative burden of glycation and metabolic stress^56,57^.

HDL reduces the risk for cardiovascular diseases by absorbing cholesterol from the blood and carrying it to the liver for processing, thereby decreasing the accumulation of atherosclerotic plaques within the wall of blood vessels^58^. With aging, both the quantity and especially the quality and function of HDL decline^59^. Aging alters HDL composition, impairs its antioxidant capacity, and reduces its protective effects against atherosclerosis and inflammation^60^. Low or dysfunctional HDL is associated with increased risk of age-related diseases and reduced longevity^61^.

High levels of triglycerides indicate obesity and metabolic syndrome and are risk factors for heart disease and stroke^62,63^. Furthermore, elevated triglyceride levels are significantly associated with accelerated biological aging, frailty, and increased all-cause mortality^64–66^.

ALT is an enzyme primarily found in the liver and plays a critical role in amino acid metabolism and metabolic health^67^. Elevated ALT levels can indicate liver damage and are often associated with conditions such as fatty liver disease, hepatitis, or excessive alcohol consumption^68–70^. Paradoxically, ALT levels decrease with age, especially in frail individuals. Lower ALT in older adults is associated with reduced hepatic metabolic activity, decreased muscle mass, and increased frailty, all of which are features of accelerated biological aging. Low ALT is a predictor of reduced survival, likely reflecting diminished organ reserve and resilience^71,72^.

Albumin is the most abundant blood plasma protein synthesized by the liver,. Lower albumin levels can indicate liver or kidney disease, as well as inflammation or infections, and are a robust marker of frailty. By contrast, high albumin levels indicate dehydration, but are also in well-hydrated individuals, associated with younger biological age and better health outcomes^73–77^.

**Fig. 4:**
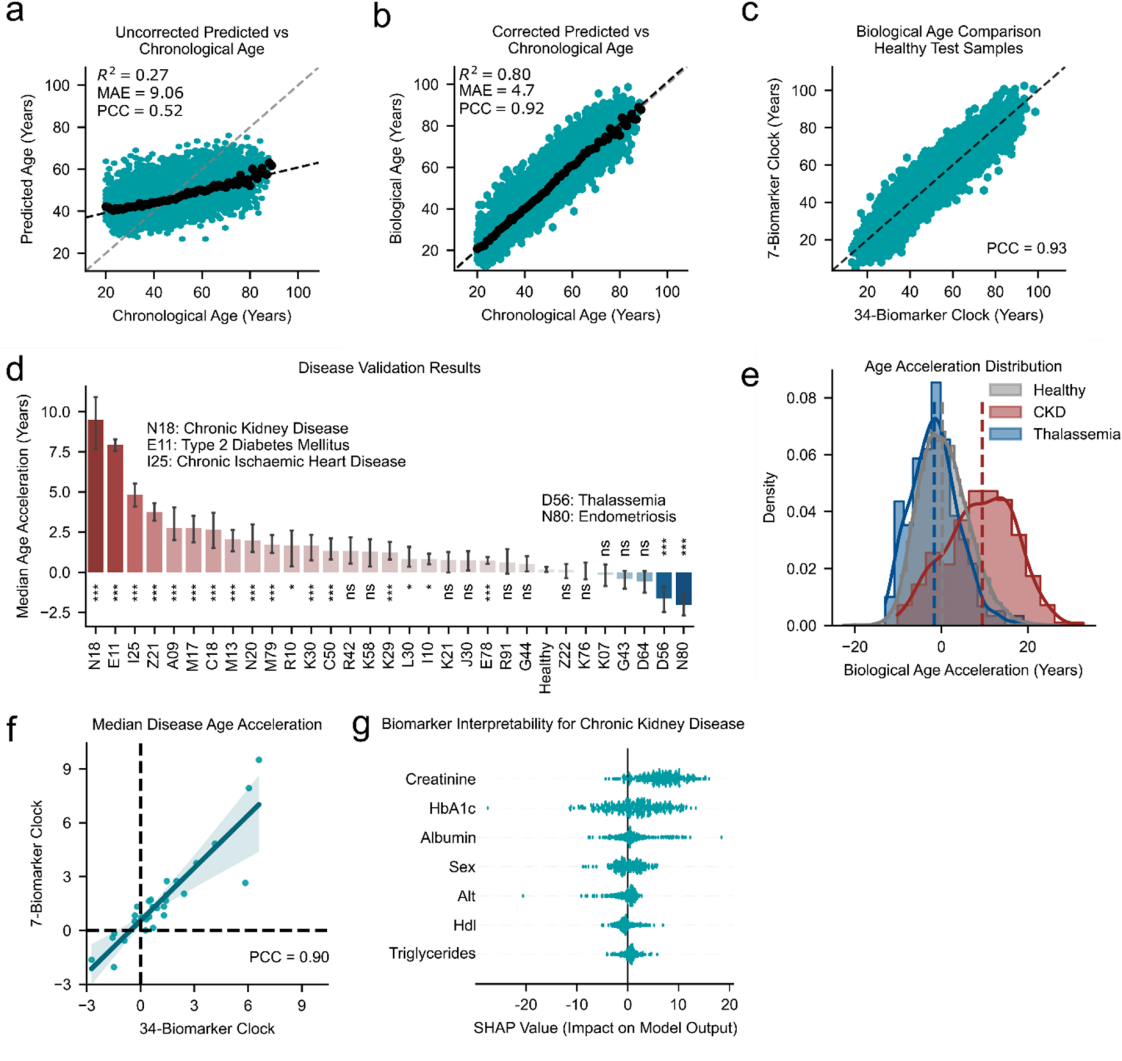
Performance and application of the 7-biomarker aging clock. **(a)** Predicted age vs. chronological age using an MLP model trained on seven features: creatinine, HbA1c, albumin, ALT, HDL, triglycerides, and sex. R^2^: 0.27, MAE: 9.06 years, PCC: 0.52. (*black* *dots*) show the median predicted age for healthy individuals per chronological age bin. **(b)** Biological age prediction after skew correction: R^2^: 0.80, MAE: 4.7 years, PCC: 0.92. (*black dots*) show the median predicted age for healthy individuals per chronological age bin. **(c)** Comparison of biological age estimates between the 34-biomarker and the 7-biomarker clocks in healthy individuals from the test dataset. A strong linear relationship is observed (PCC = 0.93). **(d)** Median age acceleration for chronic diseases for the 7-biomarker aging clock. Most chronic diseases result in an increased predicted biological age, with N18 (chronic kidney disease) resulting in the largest increase. D80 (Endometriosis) resulted in the strongest reduction in predicted age. Bars represent medians ± 95% CI. Statistical comparisons were performed using the Mann-Whitney U test, with Bonferroni correction applied for multiple comparisons. All comparisons were conducted against the healthy test group. Adjusted P values are annotated: ns (not significant), *: *P* < 0.05, **: *P* < 0.01, ***: *P* < 0.001 **(e)** Age acceleration distribution for healthy controls (*grey color*), thalassemia (*blue color*), and chronic kidney disease (CKD, *red color*) for the 7-biomarker aging clock. **(f)** Correlation of median age acceleration across ICD-coded disease groups between the 7-biomarker and the 34-biomarker clocks. PCC: 0.9. **(g)** Feature importance for individuals with chronic kidney disease based on a modified, age-matched SHAP approach. This method explains individual predictions relative to an age-matched healthy background, reducing age confounding and enhancing biological interpretability. Creatinine had the largest impact on model output.

Interestingly, the model identified sex as an important variable for predicting biological age. Our 7-biomarker uncorrected first-generation clock showed an R^2^ of 0.27, with a MAE of 9.06 years, and a PCC of 0.52 (Fig. 4a). After correction of the skewness, the R^2^ improved to 0.8 and the MAE to 4.7 years, resulting in a PCC of 0.92 (Fig. 4b). The corrected predictions of the 34-biomarker clock and the 7-biomarker clock are strongly correlated (Fig. 4c, PCC: 0.93).

Similarly, we observed that this 7-biomarker clock reliably distinguishes age acceleration in the same chronic diseases (Fig. 4d, e) and replicates the pattern observed with our 34-biomarker clock (Fig. 3f, g). To validate that the 7-biomarker clock captures the disease age accelerations, we compared the median age acceleration per disease of both clocks. We found a strong positive correlation (PCC:0.9; Fig. 4f), confirming that the 7-biomarker clock captures the same age-related and disease-associated information as the 34-biomarker clock. This result demonstrates that robust biological age predictions can be achieved with only six routinely measured clinical biomarkers and sex, substantiating the utility of our clock as an efficient and more cost-effective alternative for a biological age assessment.

To interpret the 7-biomarker clock’s predictions for disease-related age acceleration and to identify the key biomarkers driving these results, we applied a modified local SHapley Additive exPlanations^78^ (SHAP) approach, designed to provide age-contextualized interpretations (see Methods for details). Specifically, each individual was interpreted in relation to an age-matched background population of healthy individuals, enabling locally accurate explanations and mitigating the confounding effects of age. As a case study, we analyzed the modified SHAP values for individuals with chronic kidney disease (Fig. 4g). SHAP values provide insights into the output of machine learning models by quantifying the impact of each feature or biomarker on the model’s prediction^78^. This method allowed us to interpret the model’s learned associations, showing which biomarkers contribute most to the prediction output for chronic kidney disease (Fig. 4g). Creatinine showed the strongest contribution to the predicted age acceleration in chronic kidney disease patients, aligning with the established role of creatinine as a clinical marker of kidney function and age-related decline in renal function^52^. Importantly, although our clock was trained exclusively on healthy individuals with no kidney disease samples included, it still prioritized creatinine, demonstrating its ability to generalize key biomarker associations to new, unseen conditions. This result strongly reinforces the utility of the 7-biomarker clock in capturing meaningful aging-related information that remains relevant across different health states.

**Fig. 5:**
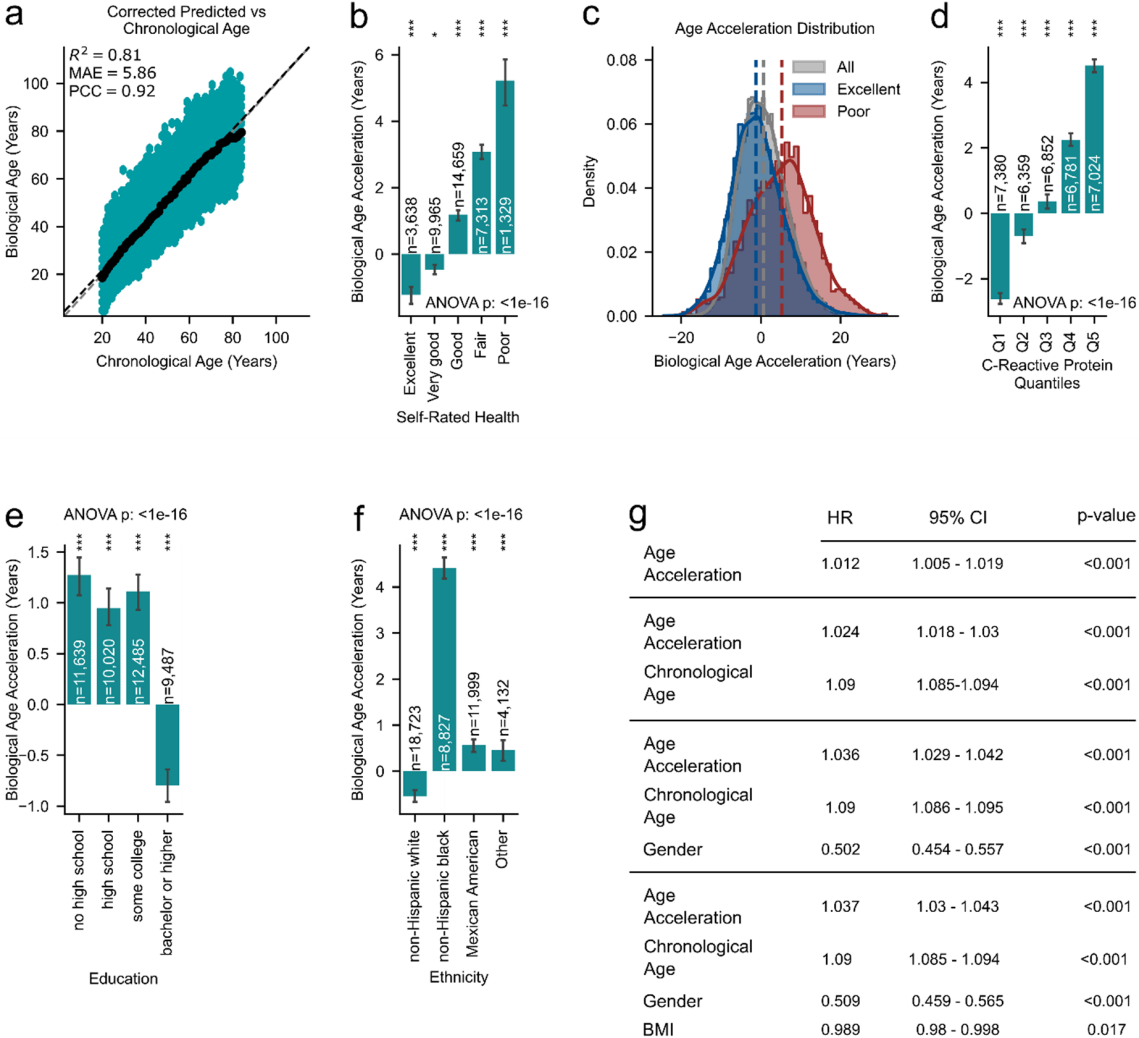
Validation of the 7-biomarker clock in the NHANES cohort. **(a)** Scatter plot showing chronological age (x-axis) versus skew-corrected predicted biological age (y-axis) from the 7-biomarker clock in the NHANES validation cohort. (*black dots*) indicate the median predicted age for each chronological age group. **(b)** The 7-biomarker clock predicts self-reported health outcomes in the NHANES dataset. Individuals reported as having poor health showed the greatest positive age acceleration, while those reported as having excellent health were predicted to be younger. ANOVA: *P* < 1e-16. **(c)** Age acceleration distribution for all individuals (*grey color*), poor self-reported health (*red color*), and excellent self-reported health (*blue color*) for the 7-biomarker aging clock based on the NHANES dataset. **(d)** Age-acceleration across CRP quantiles (Q1 = lowest inflammation, Q5 = highest). Higher chronic-inflammation burden is associated with greater biological-age acceleration. **(e)** Lower education levels show biological age acceleration, which is absent in higher education (Bachelor’s degree and higher). ANOVA: *P* < 1e-16. **(f)** Association between ethnicity and biological age acceleration, with non-Hispanic Blacks showing accelerated biological aging. ANOVA: *P* < 1e-16. **(g)** Hazard ratio analysis for biological age acceleration and implications for mortality prediction. Four Cox proportional hazard models were used to assess the predictive power of the biological age acceleration of the 7-biomarker clock. Model 1 included only the biological age acceleration, while Models 2–4 subsequently adjusted for chronological age, gender, and BMI. Hazard ratio values with a 95% confidence interval (CI) are shown. **b**,** d**,** e**,** f)** Two-sided Wilcoxon signed-rank test against a zero-median with Bonferroni correction. Bars represent medians ± 95% CI; numbers above bars show group sizes. ns: non-significant, *: *P* < 0.05, **: *P* < 0.01, ***: *P* < 0.001.

To further evaluate the robustness of the 7-biomarker clock, we assessed its performance on the NHANES dataset (see Methods for details), which features a distinct ethnic distribution compared to the training data. The 7-biomarker clock showed only a small median systematic bias of 0.66 years (Fig. 5a). The biological age acceleration in relation to self-reported health showed a clear pattern, with excellent self-reported health associated with an average age deceleration and poor self-rated health corresponding to the highest age acceleration (Fig. 5b, c, ANOVA *P* < 1e-16, see Supplementary Table 1 for statistics). This pattern supports the validity of our biological aging clock, as it demonstrates its sensitivity to subjective health status.

To examine the role of inflammation on biological aging, we stratified individuals in the NHANES dataset by quantiles of circulating C-reactive protein (CRP) levels and assessed biological age acceleration. A clear trend was observed: individuals in the lowest CRP quantiles (Q1 and Q2) exhibited significant age deceleration, while those in the upper quantiles (Q3 - Q5) showed progressively higher age acceleration, with Q5 displaying the greatest increase (Fig. 5d, ANOVA *P* < 1e–16, see Supplementary Table 1 for statistics). These findings demonstrate the sensitivity of the 7-biomarker clock to inflammatory status, despite CRP not being among its predictors. This underscores the clock’s significant potential as a holistic indicator of biological aging.

Educational attainment has been linked to various health outcomes, with higher levels often associated with better health and slower aging^79,80^. We observed that individuals with lower educational levels exhibited similar biological age accelerations, which were higher compared to those with a bachelor’s degree or higher (Fig. 5e, ANOVA *P* < 1e-16, see Supplementary Table 1 for statistics). In line with previous work^81^, our results suggests that higher education may be associated with a slower biological aging process as measured by the 7-biomarker clock.

Ethnicity has been shown to strongly affect aging clock predictions^37,38^ and potentially the aging process itself, with research suggesting that ethnic disparities may be influenced by factors such as access to healthcare, socioeconomic status, and genetics^82,83^. In our predictions, non-Hispanic Black individuals showed the strongest age acceleration (Fig. 5f ANOVA *P* < 1e-16, see Supplementary Table 1 for statistics). This could potentially be due to the composition of our training dataset, which was predominantly based on Southeast Asian populations (Fig. 1b, Supplementary Fig. 1b). The underrepresentation of non-Asian (Fig. 1b, Supplementary Fig. 1b) individuals in the training dataset may contribute to the observed discrepancy. Conversely, non-Hispanic White healthy individuals showed an average biological age prediction that aligned closely with their chronological age (Fig. 5e), suggesting that the clock performs well for this group.

To assess the predictive power of biological age acceleration for mortality (excluding accidental deaths, see Methods for details), we conducted a hazard ratio (HR) analysis across four models (Fig. 5g). The first model included only the biological age acceleration value, while the subsequent models were adjusted for chronological age, gender, and BMI. In all models, age acceleration predicted with the 7-biomarker clock consistently showed a significant positive HR, ranging from 1.012 in the first model to 1.037 in the fully adjusted model. Chronological age was in all models significantly and positively associated with mortality, indicating that aging is a major risk factor for disease^5^, while female gender showed a significant negative association. BMI exhibited a weak negative association with mortality. These results demonstrate that the biological age acceleration predictions from our 7-biomarker clock are reliable and robust indicators of health risk, even though the clock was not trained on mortality data. Importantly, NHANES serves as an external validation cohort with a different ethnic distribution, suggesting that our 7-biomarker clock maintains accuracy and improves age-gap predictions across diverse population subgroups.

To benchmark the performance of our 7-biomarker clock against established mortality-trained clocks, we applied our clock, along with the published PhenoAge^17^ and Klemera-Doubal Method^33^ (KDM) clocks, to the NHANES dataset and compared both predicted ages and survival stratifications (Supplementary Fig. 3). Our clock’s predicted biological ages showed a strong correlation with both PhenoAge (PCC = 0.94; Supplementary Fig. 3a) and KDM (PCC = 0.78; Supplementary Fig. 3b). We further stratified individuals into the top and bottom 25% of biological age acceleration for each clock and performed survival analysis. For both females and males, our clock significantly differentiated survival outcomes (Supplementary Fig. 3c, d), despite not being trained on mortality rates. In females, PhenoAge, trained specifically to predict mortality, showed a stronger separation. In males, our clock showed comparable or even slightly improved stratification for the 25% individuals with the highest age acceleration compared to PhenoAge. KDM failed to significantly predict mortality in either sex.

**Fig. 6:**
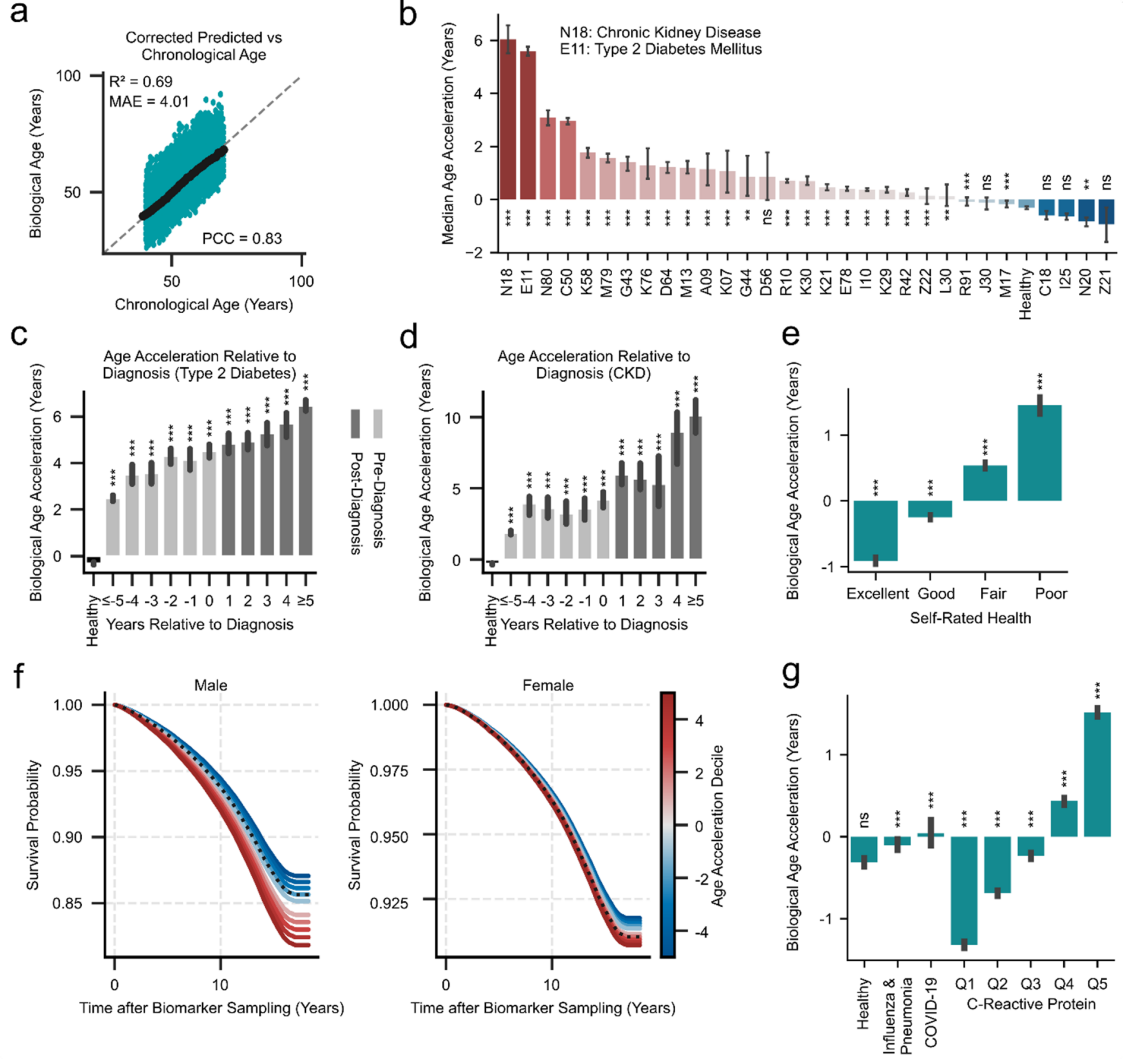
Validation of the 7-biomarker clock in the UK Biobank cohort. **(a)** Scatter plot showing chronological age (x-axis) versus skew-corrected predicted biological age (y-axis) from the 7-biomarker clock in the disease-free UK Biobank validation cohort. (*black dots*) indicate the median predicted age for each chronological age group. **(b)** Median age acceleration for chronic diseases for the 7-biomarker aging clock. Chronic diseases result in an increased predicted biological age. Bars represent medians ± 95% CI. Statistical comparisons were performed using the Mann-Whitney U test, with Bonferroni correction applied for multiple comparisons. All comparisons were conducted against the healthy test group. **c-d)** Longitudinal analysis. Biological-age acceleration relative to the year of first diagnosis for (**c**) type 2 diabetes and (**d**) chronic kidney disease (CKD). Individuals already diagnosed at the time of blood draw, as well as those who developed the disease up to 5 years later, showed significantly increased biological age acceleration compared to healthy controls (P values from Mann-Whitney U tests). Bars represent medians ± 95% CI. **e)** The 7-biomarker clock predicts self-reported health outcomes in the UK Biobank dataset. Individuals reported as having poor health showed the greatest positive age acceleration, while those reported as having excellent health were predicted to be younger. ANOVA: *P* < 1e-16 Two-sided Wilcoxon signed rank-test against a zero-median with Bonferroni correction. Bars represent medians ± 95% CI. **f)** Kaplan–Meier survival analysis in females and males stratified by biological age acceleration into deciles (*blue color* = decelerated, *red color* = accelerated). Biological age acceleration is strongly predictive of survival in both sexes (Cox proportional hazards model with chronological age as covariate; *P* < 1e-16). **g)** Robustness to inflammation and acute infections. Median age-acceleration for healthy individuals (*n* = 47,712), patients diagnosed with J09–J18 (influenza & pneumonia; *n* = 30,065) or U07.1 (COVID-19; *n* = 7,137), and individuals stratified into CRP quantiles Q1–Q5 (*n* ≈ 84,000–87,000 per group). CRP quantiles represent population-wide quintiles of C-reactive protein levels, with Q1 denoting the lowest 20% and Q5 the highest 20%. Bars represent medians ± 95% CI. Wilcoxon signed-rank tests were performed against a null hypothesis of zero acceleration. Adjusted P values are annotated: ns (not significant), *: *P* < 0.05, **: *P* < 0.01, ***: *P* < 0.001.

To further validate the generalizability and clinical relevance of our 7-biomarker clock, we applied it to the UK Biobank dataset, comprising biomarker and all-cause mortality data from over 500,000 individuals (see Methods). The predicted biological age showed a significant correlation with the chronological age among healthy individuals (PCC = 0.83), with a MAE of 4.01 years and a systematic median bias of -0.31 years (Fig. 6a).

Building on this, we next assessed the median biological age acceleration across a range of chronic diseases and found consistently elevated values in individuals diagnosed with type 2 diabetes (E11) and chronic kidney disease (N18), both of which previously exhibited the highest age acceleration (Figs. 3f and 4d and e). The UK Biobank data replicated these findings, with individuals carrying E11 or N18 ICD-10 codes displaying among the strongest biological age acceleration estimates in the entire disease panel (Fig. 6b). Although differences in effect size were observed, potentially due to population-specific variation and/or treatment differences, the results confirm the generalizability of the 7-biomarker clock across multiethnic populations. Notably, healthy individuals consistently exhibited negligible or negative age acceleration, further supporting the clinical relevance of the clock.

To evaluate whether biological age acceleration preceded disease diagnosis, we examined longitudinal age gaps in individuals who later developed chronic conditions. As a first step, we asked whether individuals not yet diagnosed with a given disease at the time of biomarker sampling, but who developed the disease at any later time, already showed biological age acceleration. Indeed, across multiple chronic diseases, individuals later diagnosed showed significantly elevated predicted biological ages compared to healthy controls, despite lacking a clinical diagnosis at the time of sampling (Supplementary Fig. 4a). To further investigate the temporal resolution of this early signal, we next assessed biological age acceleration as a function of time to diagnosis. Strikingly, individuals who later developed type 2 diabetes (Fig. 6d) or chronic kidney disease (Fig. 6e) already exhibited a significantly elevated biological age acceleration up to five years prior to diagnosis, compared to healthy controls (Bonferroni-adjusted *P* < 0.001). To corroborate these results and account for potential inaccuracies in ICD annotation timing, we repeated the diabetes analysis with an additional filter: individuals had to have an HbA1c value < 6.5% at the time of biomarker sampling, excluding pre-existing diabetes according to standard guidelines^84^. Notably, even under this stricter definition, individuals who were later diagnosed with diabetes still exhibited significant biological age acceleration at the time of sampling (Supplementary Fig. 4b). These results suggest that our 7-biomarker clock detects early, preclinical aging signatures that precede and potentially forecast future disease onset.

To assess whether such signals also reflect more subjective evaluations of health in the UK Biobank, we examined biological age acceleration in relation to self-reported health status. Biological age acceleration in relation to self-reported health showed a clear pattern, similar to the NHANES data (Fig. 5b), with excellent self-reported health being associated with an average age deceleration and poor self-rated health corresponding to the highest age acceleration (Fig. 6e, ANOVA *P* < 1e-16, see Supplementary Table 1 for statistics).

We next asked whether biological age acceleration also predicts long-term outcomes by testing its association with all-cause mortality. Stratifying participants by age acceleration quantiles, we observed a monotonic decline in survival probability with increasing acceleration for both females and males (Fig. 6f, see Supplementary Table 1 for statistics). Individuals with the highest biological age acceleration had significantly reduced survival compared to those with negative acceleration. These survival curves illustrate that the biological age gap predicted by our 7-biomarker clock is a powerful and sex-independent predictor of all-cause mortality.

To further probe the biological relevance and robustness of our clock, we examined age acceleration across CRP quantiles in the UK Biobank. Similar to the trend we observed in the NHANES dataset (Fig. 5d) individuals in the lowest CRP quantiles showed significant age deceleration, while those in the highest quantiles exhibited increasingly pronounced age acceleration (Fig. 6g, ANOVA *P* < 1e–16, Supplementary Table 1). Although CRP is not a component of the clock, these findings highlight its sensitivity to chronic low-grade inflammation. Aging clocks are known to be confounded by acute inflammation and transient physiological stressors, which can temporarily alter biomarker profiles and inflate biological age estimates^85–87^. To assess whether our clock, despite capturing inflammation-related signals, remains robust to acute perturbations, we examined biological age acceleration in individuals with ICD codes for acute respiratory infections, i.e., influenza or pneumonia (J09–J18) and COVID-19 (U07.1). Both groups showed median biological age accelerations of -0.10 and + 0.04 years, respectively, suggesting that acute disease states do not substantially bias the clock (Fig. 6g). Together, these findings support the specificity of the 7-biomarker clock for long-term physiological changes and its robustness to short-term and transient inflammatory events.

## Discussion

Quantifying aging through biological clocks is essential for identifying tipping points towards chronic and age-related diseases^51^. In the present study, we trained biological clocks using clinical biochemistry markers collected over twenty years from 59,741 healthy Southeast Asian samples and identified 34 markers predicting biological age. To improve the prediction of the age gap associated with disease, we developed a novel normalization approach that eliminates the generally observed skewness of first-generation aging clocks. Furthermore, we demonstrated that the number of biomarkers could be systematically reduced to a minimal set of 7 without compromising the predictive performance of the biological aging clock. We showed that both sex-adjusted aging clocks, namely the comprehensive 34-biomarker model and the 7-biomarker model, exhibit a high degree of translational potential, accurately predicting biological age and detecting disease-associated age acceleration. To establish the generalizability of the 7-biomarker clock, we rigorously validated it using data from NHANES and the UK Biobank.

We evaluated the performance of both the comprehensive 34- and the 7-biomarker aging clocks to assess their accuracy and explanatory power in predicting biological age. The interpolated median-corrected 34-biomarker clock achieved an MAE of 5.3 years. The regression model yielded an R^2^ of 0.74 between the corrected predicted age and chronological age, indicating a strong alignment after bias correction. Similarly, the 7-biomarker clock retained high accuracy with an R^2^ of 0.8. However, it is essential to emphasize that a perfect R^2^ score of 1 with an MAE of 0 would be meaningless in the context of biological aging, as it would indicate a model that solely predicts chronological age without capturing any biological variation. For instance, if a model predicted only the mean age value for all samples, i.e., a flat line without variance, and were corrected with our method, it would yield an artificially perfect chronological age model and consequently lack the critical biological information necessary for meaningful interpretation. Our correction method, therefore, is only effective when the uncorrected model predictions have already captured biological information. Importantly, our correction method uses interpolated medians computed only from the training data to align predicted ages with the line of identity, thereby preventing data leakage. Notably, this approach is highly robust, as the skew we observed is consistent across the training, test, and validation datasets, highlighting its applicability and reliability.

One potential cause of the skew is the selection bias inherent in human datasets, where biologically older individuals are underrepresented due to higher mortality rates. Specifically, as chronological age increases, biologically older individuals are more likely to have died and thus are absent from the dataset, a classical survivor bias^20^. This results in a dataset where, at older chronological ages, the remaining individuals are disproportionately biologically younger, leading the model to predict these older chronological age individuals as biologically younger than their actual age. To address this concern, in our earlier work, we implemented a different correction method in a transcriptomic *C. elegans* clock^20^. This method accounted for the reduced representation of biologically older individuals by leveraging comprehensive lifespan data from isogenic *C. elegans* populations, thereby mitigating the impact of selection bias and improving prediction accuracy. However, applying such a correction in human datasets poses significant challenges due to the lack of detailed lifespan information and the inherent genetic and environmental diversity among human populations.

Another contributor to the observed prediction skew could be regression to the mean, whereby models trained to predict chronological age tend to compress predictions toward the age distribution’s central values^32,88^. This results in younger individuals being predicted as older, and older individuals being predicted as younger. Importantly, despite the skew, our models capture biologically meaningful differences; for example, individuals with type 2 diabetes are consistently predicted to be older than healthy controls of the same chronological age. However, the systematic skew distorts the predicted age scale and hinders interpretability, particularly when comparing across age groups. The rationale for applying this skew correction is, therefore, biological in nature: it enables a more interpretable and consistent biological age prediction and ensures that deviations from chronological age reflect meaningful biological signals rather than systematic model bias. By centering predictions along the identity line, our correction restores a consistent interpretative framework, allowing age deviations to be evaluated relative to an unbiased reference and facilitating meaningful comparisons across individuals and groups.

Most first-generation aging clocks based on blood biochemistry were primarily validated on their ability to predict chronological age and reported MAE and R^2^ values as their main performance metrics, with MAEs ranging from ~ 5.5 to ~ 7 years^35–37,89–92^. While this comparison places our clocks on top of these first-generation aging clocks, it is important to note that our primary focus is not on chronological age prediction but on the extraction of biologically meaningful information. Second-generation clocks aim to predict outcomes such as mortality or time to death and incorporate chronological age during their training process. We also used chronological age to guide the correction process, thereby resembling more of a pseudo-second-generation clock. A key strength of our approach is that we built a biological aging clock without relying on mortality data for training and demonstrated its ability to track disease risk and mortality outcomes through extensive biological validations in independent cohorts, including the UK Biobank and NHANES.

To identify the most informative biomarkers, we employed an iterative feature reduction strategy based on an adapted SHAP framework that accounted for local age group contributions. While SHAP values are increasingly used to interpret complex models, their application in aging research presents unique challenges. Specifically, the default SHAP approach explains predictions relative to a global baseline^78^, which may obscure biologically meaningful deviations in age-related phenotypes. To address this, we implemented a modified SHAP framework using locally age-matched healthy individuals as background data for each explanation. This approach enabled context-specific interpretations and minimized age as a confounding variable, a critical adjustment in the context of biological aging. Additionally, we applied a correction offset to align explanations with deviations from the individual’s chronological age, rather than from the global model mean. To the best of our knowledge, this combined strategy of local SHAP explanations with age-matched background and chronological-age alignment has not been systematically applied before, and may serve as a generalizable approach for interpreting personalized biomarker models in aging and beyond.

Despite the reduction in biomarker inputs, the 7-biomarker clock replicates key findings from the 34-biomarker clock, including consistent age acceleration in chronic diseases such as diabetes and chronic kidney disease. Importantly, the high correlation between the corrected predictions of both clocks and the strong agreement in median age accelerations underscore the capability of the 7-biomarker clock to capture the same biological aging signals.

We showed that both biological age clocks predict an increased biological age across a wide range of ICD-10 codes in our Southeast Asian dataset. The 7-biomarker clock replicated the results of the comprehensive 34-biomarker clock, indicating that even with reduced biomarker input, biologically meaningful age accelerations can be robustly detected across multiple disease states (Figs. 3f and 4d; Supplementary Table 1). Importantly, validation in the UK Biobank confirmed the generalizability of these results (Fig. 6c). Chronic kidney disease and type 2 diabetes again ranked among the top age-accelerated conditions, showing consistent trends across both cohorts. This cross-population reproducibility demonstrates the robustness of our clock across ethnicities and healthcare contexts.

However, several diseases showed divergent patterns between the Southeast Asian and UK Biobank cohorts. For example, thalassemia (D56) and endometriosis (N80) were associated with a significantly younger predicted age in the Southeast Asian dataset, but not in the UK Biobank, where thalassemia showed no significant effect and endometriosis ranked among the most age-accelerated conditions. Similarly, while Z21 (HIV-positive status) was age-accelerated in the Southeast Asian cohort, it showed no significant effect in the UK Biobank. These discrepancies may reflect differences in comorbidity patterns, treatment practices (e.g., blood transfusions), or demographic characteristics, and highlight the importance of cohort context in biological age modeling.

Ethnic differences in blood biochemistry^93^ and aging clock predictions^37,38^ are well-documented and underscore the importance of cross-population validation for ensuring generalizability. Our 7-biomarker clock was trained primarily on a Southeast Asian population, whereas both NHANES and the UK Biobank predominantly represent Caucasian populations. Remarkably, our 7-biomarker clock showed only a minimal systematic bias when applied to the NHANES (0.66 years) and/or UK Biobank (-0.31 years) dataset, indicating robust performance across different ethnic groups. Interestingly, one of the most important features in our clock, creatinine, is known to exhibit population-specific distributions^93^. Southeast Asian women have significantly lower average creatinine levels compared to Caucasian standards, which may partially explain the observed bias^93^. Despite this, the age acceleration values demonstrated expected health patterns: individuals with poor self-reported health exhibited positive age accelerations, while those with excellent self-reported health showed negative accelerations on average. These findings highlight the potential of our clocks as a universal tool for population-wide health monitoring while also emphasizing the need to consider population-specific biochemical differences when applying aging clocks across diverse groups.

It has been observed that in most elderly, pro-inflammatory biomarkers are elevated, reflecting a chronic low-grade inflammatory state often referred to as inflammaging^94–97^. The senescence-associated secretory phenotype contributes to this process by altering the tissue microenvironment^98^ and promoting pathological changes^99^. Consequently, many aging clocks are confounded by inflammation and predict elevated biological ages during acute infections, because inflammation tends to increase with age, these models learn to associate higher inflammatory levels with older biological age^86,87^. This is problematic in clinical contexts, as acute infections can transiently elevate inflammatory markers even in otherwise healthy individuals, leading to increased biological age predictions. These inflammation-induced shifts are typically reversible upon recovery and do not reflect true biological aging^85^.

To assess whether our 7-biomarker clock is similarly affected, we directly examined age acceleration across CRP quantiles, a well-established marker of inflammation. In both NHANES and the UK Biobank, we observed a clear and graded relationship: individuals in the lowest CRP quantiles showed significant age deceleration, while those in the highest quantiles displayed progressively greater age acceleration. Notably, CRP was not included in our model, yet its correlation with age acceleration affirms the clock’s sensitivity to chronic low-grade inflammation. Crucially, individuals diagnosed with acute respiratory infections, including influenza, pneumonia (J09–J18), or COVID-19 (U07.1), did not exhibit substantially elevated biological age accelerations, despite typically showing high CRP values during acute illness. This indicates that the clock is not biased by transient immune activation. Unlike models that include volatile inflammatory biomarkers such as CRP, which exhibit high temporal variability^100,101^ and may distort age predictions, our clock focuses on stable clinical chemistry biomarkers. This design enables robust biological age estimates, even in the presence of temporary immune or inflammatory perturbations.

Our results reveal intriguing possibilities for the broader applications of biological age clocks in healthcare and aging research. Blood-based biological age clocks could demonstrate significant utility as key clinical measures in preventive medicine. As such, routine biological age assessments every 6–12 months could help detect early signs of disease development (i.e., type 2 diabetes mellitus, etc.), enabling timely and personalized interventions and treatment strategies, and lifestyle modifications to slow progression, improve health outcomes, and potentially prevent the onset of age-related diseases. Bridging or lowering the biological age acceleration has consistently been shown to be associated with fewer comorbidities^102^, the prevalence of age-related diseases, and increased musculoskeletal and mental health compared to age-matched controls^89,91,103^. Our 7-biomarker clock, with minimal ethnic bias, uses clinical biomarkers that are routinely measured in standard healthcare settings, making it both cost-effective and scalable. This accessibility makes it an ideal tool for clinicians worldwide, providing a practical model for identifying age-related disease trajectories and supporting preventative medicine.

To support real-world applicability, we envision three primary clinical use cases for our 7-biomarker aging clock, which we refer to as SevenAge: (1) Early screening, where biological age acceleration identifies individuals at elevated risk for age-related diseases (e.g., diabetes, chronic kidney disease) prior to clinical onset; (2) longitudinal monitoring, allowing healthcare providers to track biological aging over time and evaluate the impact of lifestyle changes, pharmacological interventions, or disease progression; and (3) treatment stratification, where patients with high biological age acceleration may benefit from more aggressive preventive strategies or closer follow-up. Because the model utilizes only routine blood markers and sex, it is highly compatible with standard health check-ups and can be seamlessly integrated into electronic health record systems. These features make the clock a practical tool for supporting a shift toward proactive, personalized aging and chronic disease management in clinical practice.

In summary, we applied a novel correction approach to overcome a generally observed skewness in first-generation aging clocks. Building on this, we employed a novel systematic iterative feature reduction strategy based on an adapted SHAP approach that accounted for local age group contributions, enabling us to reduce the required biomarkers to just six routine clinical blood biomarkers and sex. The resulting model, SevenAge, is, to our knowledge, the first clinical biological aging clock trained on a Southeast Asian population and validated across multiethnic cohorts. Looking ahead, integrating biological age assessments into clinical workflows could revolutionize how we approach aging and chronic disease management. By offering a scalable solution, SevenAge can support population-level health monitoring and targeted interventions. Ultimately, the application of biological age clocks could shift the paradigm from reactive treatment to proactive prevention, thereby extending the healthspan and improve the quality of life for individuals worldwide.

## Materials and methods

This study was determined to be ‘out of scope’ for review by the Bumrungrad International Hospital Institutional Review Board under project registration number 343-06-25 PDSK-B NS. This study adheres to the Declaration of Helsinki’s ethical standards. Furthermore, it has been conducted in compliance with the Thai Personal Data Protection Act, which was announced on May 27, 2019, and became enforceable on June 1, 2022.

### Data acquisition and filtering

Raw clinical anonymized data were collected from the research and development data warehouse of the Bumrungrad International Hospital (Bangkok, Thailand)^44^. While the full dataset encompasses the years 2000–2025, only data from 2000 to 2022 were utilized for training and testing of the biological age clock. From an expert-derived consensus list of 51 biomarkers, we employed an iterative approach to define a suitable dataset characterized by a low proportion of missing data and a high sample size, while retaining as many biomarkers as possible. After serial thresholding of the minimum number of biomarkers measured in a sample and the fraction of all samples where a biomarker was present, we obtained the filtered version of the operational data with 34 biomarkers, 171,812 samples, and 22% missing values. We further enriched the metadata of our dataset with ICD-10 codes and drug information, categorized using the WHO Anatomical Therapeutic Chemical (ATC) Classification System (https://www.who.int/tools/atc-ddd-toolkit/atc-classification), to classify each sample into diseased or healthy (Supplementary Table 1). After the incorporation of this information, the dataset consists of 59,741 healthy samples used for model training and 112,071 diseased samples, reflecting the composition expected from the operational hospital data.

Missing values were imputed using the Iterative Imputer from the scikit-learn library^104^, employing default parameters and by constraining imputed values to a minimum of 0 to prevent negative values. The top 0.5% outlier samples for each biomarker were removed. Finally, all features were normalized to a uniform scale between 0 and 1 using linear min-max scaling, as implemented in scikit-learn.

### Deep learning model

A deep learning model was implemented using PyTorch v.2.3.0^105^, and the training was done with PyTorch Lightning v.2.3.0^106^. The model parameters included the Adam optimizer with a learning rate of 5e-3, a batch size of 2048, and a mean squared error (MSELoss) loss function. Weight decay was set to 1e-4, and the model architecture was a multi-layer perceptron that comprised three layers with sizes 1,000, 100, and 1, respectively. The ReLU activation function was applied, and no dropout or normalization layers were used. The model was trained for a maximum of 2,000 steps, with progress monitored at intervals of 10 steps.

The dataset was split into training, validation, and testing subsets using a random stratified approach based on chronological age, with proportions of 60%, 20%, and 20% on the healthy cohort, respectively.

### Biological age correction

To address the observed skew in biological age predictions, several correction methods and transformations were evaluated. Skew was present in all experiments without exception, and the clock continued to provide biologically meaningful information despite these challenges.

Correction strategies included subsampling to limit the data to a maximum of 1,000 samples per chronological age year and applying the age transformations including logistic scaling and quantile normalization to a uniform distribution.

The final correction mechanism was a non-parametric, kernel-based approach. For each chronological age year in the training dataset, we computed the median predicted biological age. These median values were used to fit a smooth correction curve using scikit-learn’s KernelRidge regression with parameters alpha = 0.001 and kernel=’additive_chi2’. The additive_chi2 kernel was chosen for its ability to model non-linear relationships in sparse, non-negative data distributions. The resulting curve captures the average age-specific prediction error and provides a year-specific offset. These offsets were subtracted from the predicted biological ages in the test and validation sets, thereby re-centering predictions around the identity line without introducting data leakage.

### Model interpretability

The models were interpreted through SHAP values using the SHAP package v0.45.1^78^, specifically the shap.DeepExplainer implementation, with default parameters as provided by the official SHAP library (https://shap.readthedocs.io/en/latest/index.html*).*

To ensure biologically meaningful SHAP interpretations, we modified the standard SHAP workflow by constructing a dynamic, age-matched background set: for each individual of chronological age *x*, healthy individuals from the training set within a ± 2.5-year window ([*x*–2, *x* + 2]) were used as the background dataset. This stratification mitigates the confounding effects of age and provides more locally accurate feature attributions, an important consideration in aging research that is not standard in current SHAP applications. Additionally, we applied the median correction offset to the SHAP base value to shift explanations toward the actual chronological age rather than the model’s global prediction mean. This ensures that SHAP explanations reflect deviations from an age-matched baseline.

Each individual diagnosed with a specific disease was analyzed independently to interpret the disease-specific contributions of the patient. The additive SHAP values for all features were subsequently aggregated to provide a summary of the disease-related effects.

### Iterative biomarker reduction

To develop a simplified version of the biological aging clock, we began by systematically eliminating highly correlated biomarkers with a Pearson correlation of greater than 0.5, reducing the initial list of 34 biomarkers to 21. For correlated biomarkers, the biomarker with the highest sample count was retained. This preprocessing step reduced redundancy and ensured that subsequent SHAP value-based ranking reflected largely independent biomarker contributions. Subsequently, we performed an iterative feature selection process, in which biomarkers with the lowest impact on the model’s performance, as measured by SHAP values, were removed. In the first two steps, we excluded the five biomarkers with the weakest performance. Thereafter, the remaining biomarkers were assessed iteratively, with the lowest-performing biomarker being removed at each step. Blood cell counts were removed from the clock to reduce cost constraints.

### Disease and population validation

Disease-specific and population-level validation studies were conducted to assess the clock’s robustness. For disease-specific validation, we identified samples affected by single ICD10 codes, eliminating cases with multiple comorbidities, and analyzed the top 20 most frequent isolated conditions in the Bumrungrad International Hospital dataset. The clock was trained exclusively on healthy subjects’ data and subsequently tested on disease-specific cohorts, with the unseen healthy test cohort serving as a control group. The statistical significance of the median population predictions for each ICD10 code was evaluated using the Mann-Whitney U test implemented in SciPy v1.5.1^108^.

For broader population validation, we utilized NHANES data obtained from the BioAge v.0.1.0 R package^108^. After retaining the 21 common biomarkers, both Bumrungrad International Hospital and NHANES datasets were processed through a joint pipeline identical to the one previously described, with the sole modification being a minimum requirement of 12 biomarkers per sample for inclusion in the analysis. The validation protocol involved training the model on the Bumrungrad International Hospital’s healthy cohort data and evaluating its performance on NHANES data.

The hazard ratio was computed with the CoxPHFitter function from Python’s lifelines package v0.24.14^109^. We excluded participants who died from accidents, i.e., non-age related deaths, and participants older than 79 as the exact chronological age could not be ascertained.

#### UK biobank validation

To further evaluate the generalizability of the 7-biomarker clock beyond the Southeast Asian training population and the U.S.-based NHANES validation, we applied the model to the UK Biobank dataset via the Research Analysis Platform. Unlike NHANES, the UK Biobank provides a larger, predominantly European-ancestry cohort with extensive longitudinal data and disease diagnoses. Biomarker values were harmonized to match the preprocessing pipeline used in the Bumrungrad dataset, and only individuals with ≥ 5 of the 6 overlapping blood biomarkers were retained for analysis.

Healthy individuals were defined based on the absence of any ICD-10 diagnoses (i.e., null ICD fields). For disease-specific analyses, we selected individuals with at least one occurrence of the relevant ICD-10 code (e.g., N18 for chronic kidney disease), without excluding comorbidities. To examine age acceleration trajectories relative to disease onset, we aligned samples by diagnosis year and binned them into the following categories: ≤ −5 years, − 4 to − 1 years (per year), year 0 (diagnosis within the same year of sampling), and + 1 to + 4 years, with all later time points grouped into ≥ + 5 years.

For survival analysis, all eligible UKB participants were included. Age acceleration values were stratified into 10 bins. Survival curves were generated separately for males and females using coxph.plot_partial_effects_on_outcome from the Python’s lifelines package v0.3^109^, with chronological age included as a covariate to account for baseline mortality risk.

### Statistics

The one-way ANOVAs were computed using Python’s pingouin package v.0.3.6^110^. Two-sided independent *t*-tests were computed with Python’s scipy.stats.ttest_ind function from the SciPy package v1.5.1^107^. Two-sided Wilcoxon signed-rank tests were performed using scipy.stats.wilcoxon, and two-sided Mann-Whitney U tests using scipy.stats.mannwhitneyu.

## Electronic supplementary material

Below is the link to the electronic supplementary material.

Supplementary Material 1

Supplementary Material 2

## Data Availability

All data generated or analyzed during this study are included in this published article and its supplementary information in Supplementary Table 1. Due to legal restrictions, individual-level data from the Southeast Asian cohort cannot be shared publicly. However, aggregate statistics relevant to all main analyses (e.g., group-level age acceleration estimates, summary statistics) are provided. NHANES data were accessed through the BioAge v0.1.0 R package^108^ and used as provided by the package. UK Biobank data are available to qualified researchers upon application through the UK Biobank portal (https://www.ukbiobank.ac.uk).
