# Supplementary material for: A sex-adjusted 7-biomarker clinical aging clock for translational preventative medicine
Source: Sci Rep. 2025 Dec 10;15:43538. doi: 10.1038/s41598-025-27478-9 (PMC12695998; doi:10.1038/s41598-025-27478-9)
Supplement: Supplementary file 1 — Supplementary Material 1 [file 41598_2025_27478_MOESM1_ESM.docx]

| **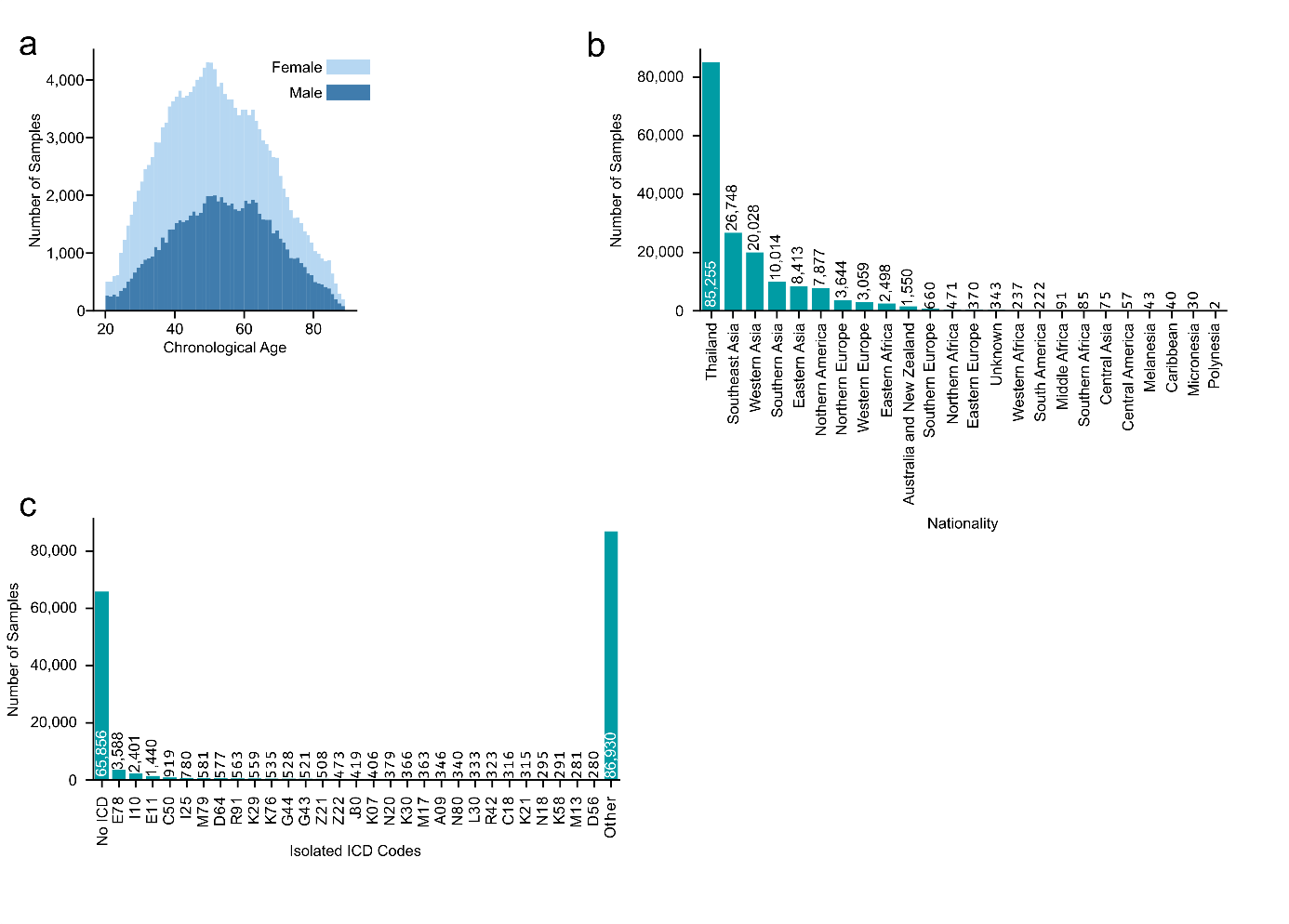** |
| --- |
| **Supplementary Figure 1.** **Demographic and clinical overview of the filtered dataset of 171,812 samples**   1. Age distribution of the samples for female and male individuals in the filtered dataset. 2. Number of samples present in the dataset for different ethnicities. The majority of samples come from Thai individuals, with other Asian regions making up the majority of the rest of the samples. 3. Frequencies of the most common diseases categorized by ICD codes. The most common recorded diseases are E78 (Lipidaemias), I10 (Essential hypertension), E11 (Type 2 diabetes mellitus), I25 (Chronic ischemic heart disease), and N18 (Chronic kidney disease). Note that we here concentrated on samples with only one ICD code, i.e., multi-morbid samples are classified as “Other”. |

| **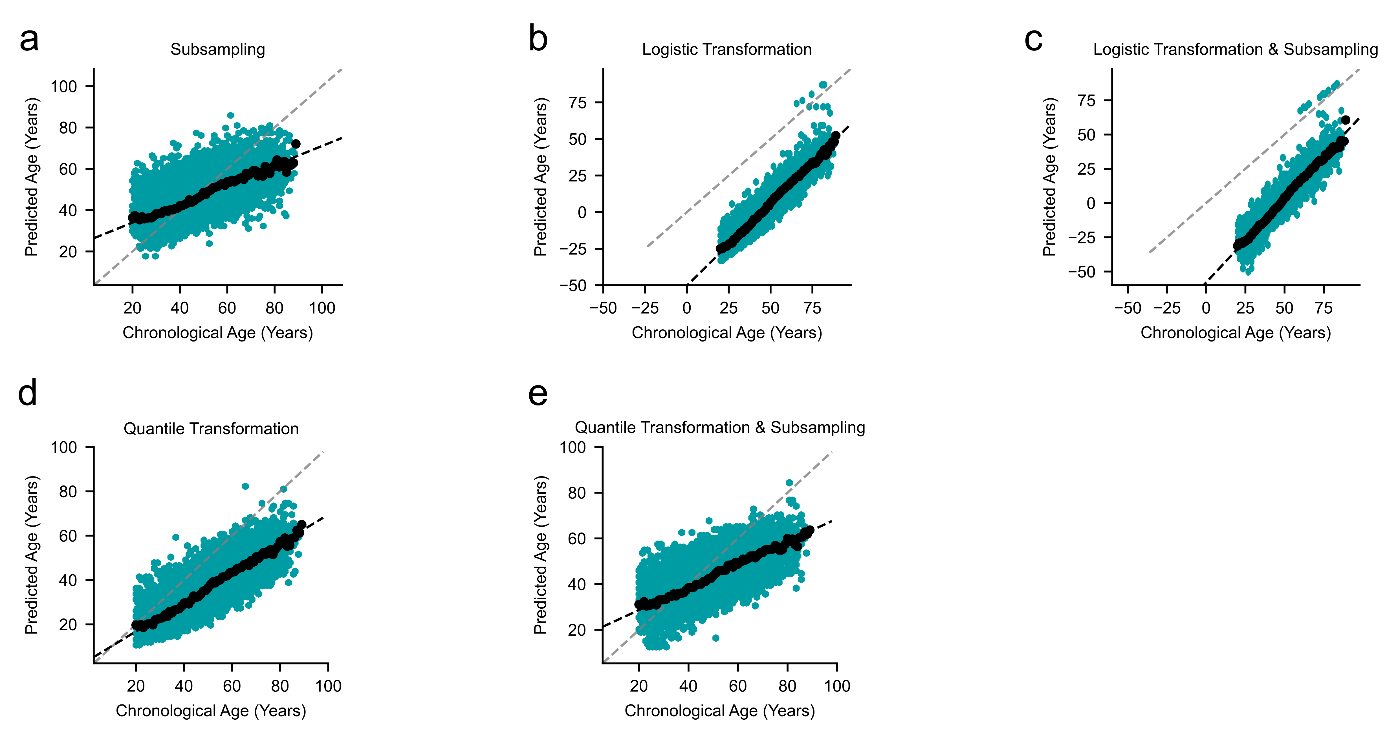** |
| --- |
| **Supplementary Figure 2. Effect of data transformations on the performance of the 34-biomarker clock**   1. Model performance after subsampling the data to a maximum of 1,000 samples per chronological year. 2. Model performance after using a logistic transformation on the chronological age. 3. Model performance after using a logistic transformation on the chronological age and subsampling the data to a maximum of 1,000 samples per age class. 4. Model performance after using a quantile transformation to a uniform distribution of the chronological age. 5. Model performance after using a quantile transformation to a uniform distribution of the chronological age and subsampling the data to a maximum of 1,000 samples per age class. |

| **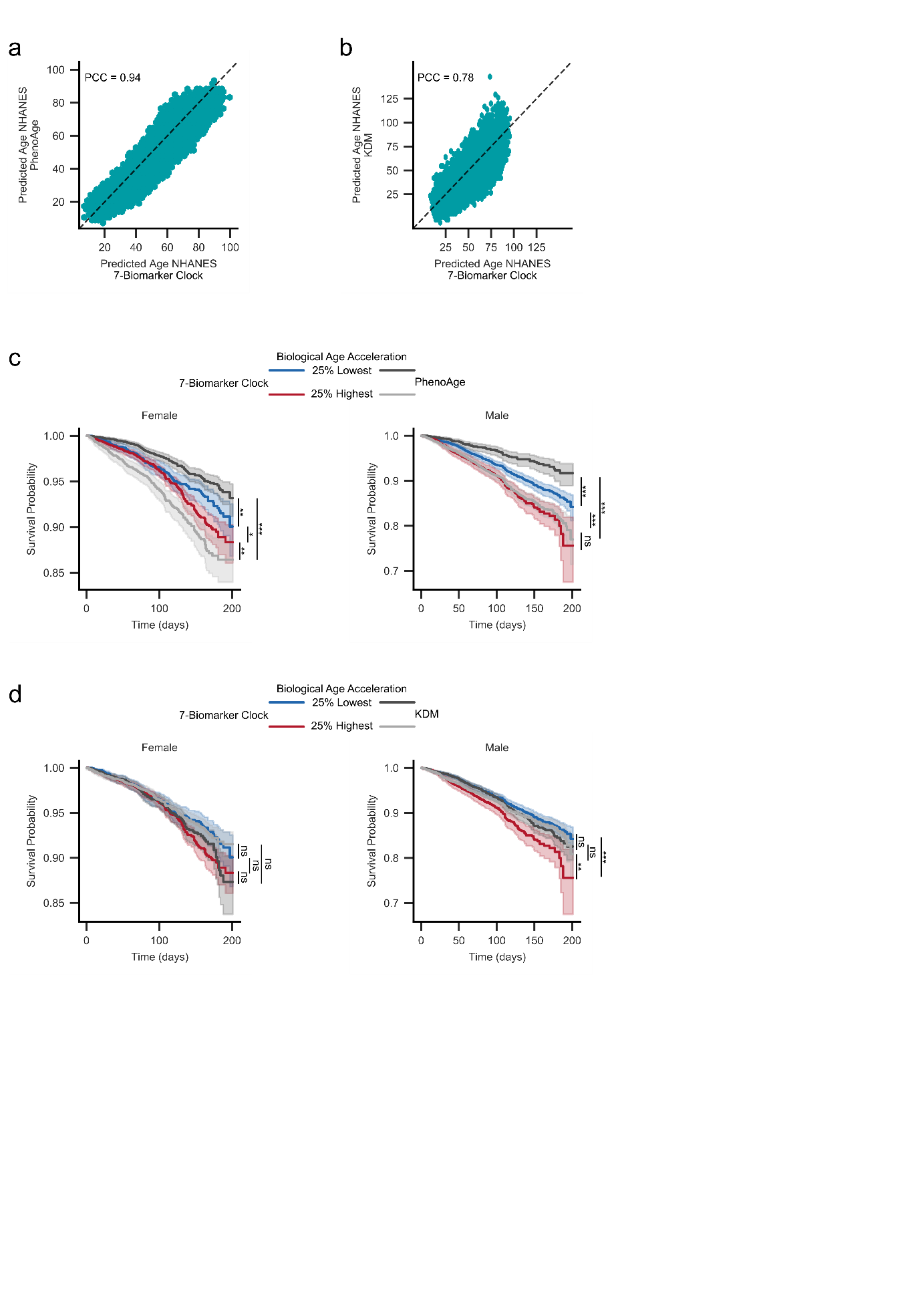** |
| --- |
| **Supplementary Figure 3. Comparison of the 7-biomarker clock with PhenoAge and KDM in the NHANES cohort. (a)** Correlation between predicted biological age from the 7-biomarker clock and PhenoAge (PCC = 0.94).  **(b)** Correlation between predicted biological age from the 7-biomarker clock and the KDM clock (PCC = 0.78). **(c)** Kaplan–Meier survival curves for female and male participants stratified by biological age acceleration (top 25%: red; bottom 25%: blue) using the 7-biomarker clock, compared to PhenoAge (gray). Significance was calculated using the log rank test. **(d)** Kaplan–Meier survival curves for female and male participants stratified by biological age acceleration as in (c), here compared to KDM (gray). Significance was calculated using the log rank test. |

| **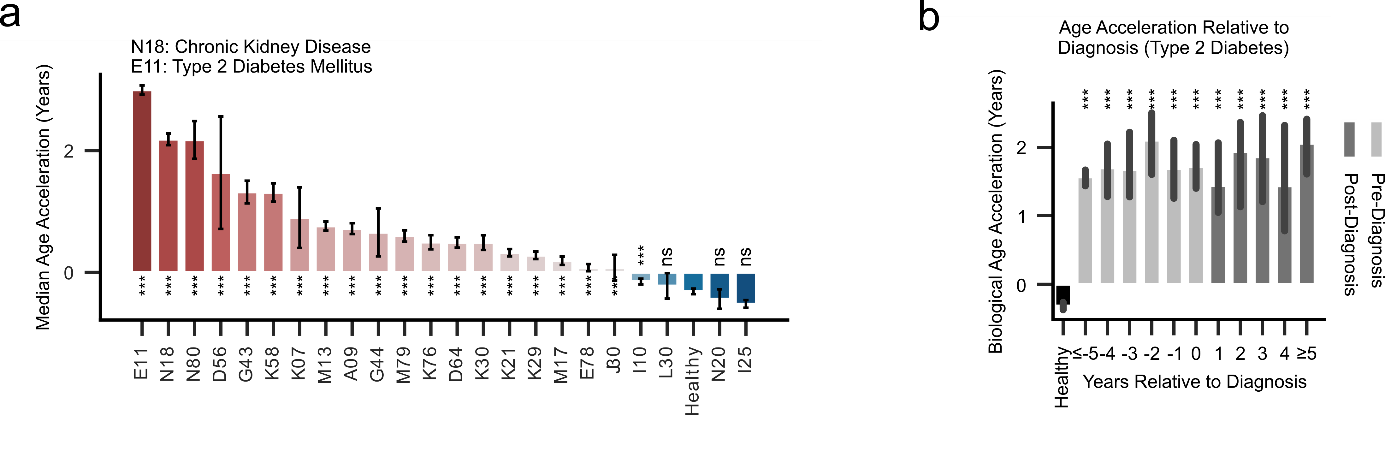** |
| --- |
| **Supplementary Figure 4. Robustness of age acceleration prior to disease diagnosis. (a)** Median age acceleration for chronic diseases for the 7-biomarker aging clock of individuals prior to clinical diagnosis. Only samples from individuals not yet diagnosed with the respective disease at the time of biomarker sampling were included. Chronic diseases were still associated with increased predicted biological ages compared to healthy controls. Bars represent medians ± 95 % CI. Statistical comparisons were performed using the Mann-Whitney U test, with Bonferroni correction applied for multiple comparisons. All comparisons were conducted against the healthy test group. **(b)** Biological age acceleration relative to time of diagnosis for individuals who were later diagnosed with type 2 diabetes (ICD E11), restricted to those with HbA1c levels <6.5% at the time of biomarker sampling (i.e., excluding individuals with pre-existing diabetes according to ADA criteria). Age acceleration remains significantly elevated up to five years before diagnosis compared to healthy controls. Bars represent median ± 95% confidence interval. Statistical comparisons were performed using Mann–Whitney U tests against the healthy group with Bonferroni correction for multiple testing. Adjusted P values are annotated: ***P < 0.001. |

M1E
